# Synchronous Teleconsultation and Monitoring Service Targeting COVID-19: Leveraging Insights for Postpandemic Health Care

**DOI:** 10.2196/37591

**Published:** 2022-12-22

**Authors:** Milena Soriano Marcolino, Clara Sousa Diniz, Bruno Azevedo Chagas, Mayara Santos Mendes, Raquel Prates, Adriana Pagano, Thiago Castro Ferreira, Maria Beatriz Moreira Alkmim, Clara Rodrigues Alves Oliveira, Isabela Nascimento Borges, Magda César Raposo, Zilma Silveira Nogueira Reis, Maria Cristina Paixão, Leonardo Bonisson Ribeiro, Gustavo Machado Rocha, Clareci Silva Cardoso, Antonio Luiz Pinho Ribeiro

**Affiliations:** 1 Department of Internal Medicine Medical School Universidade Federal de Minas Gerais Belo Horizonte Brazil; 2 Telehealth Center University Hospital and Telehealth Network of Minas Gerais Universidade Federal de Minas Gerais Belo Horizonte Brazil; 3 Institute for Health Technology Assessment Porto Alegre Brazil; 4 Department of Computer Science Instituto de Ciências Exatas Universidade Federal de Minas Gerais Belo Horizonte Brazil; 5 Arts Faculty Universidade Federal de Minas Gerais Belo Horizonte Brazil; 6 Telehealth Center Universidade Federal de São João Del-Rei Divinópolis Brazil; 7 Medical School Campus Centro Oeste Universidade Federal de São João del-Rei Divinópolis Brazil

**Keywords:** COVID-19, telemonitoring, remote consultation, telemedicine, primary health care, delivery of health care, telehealth, text message, mobile health, public health, remote care, digital health, usability

## Abstract

**Background:**

Although a great number of teleconsultation services have been developed during the COVID-19 pandemic, studies assessing usability and health care provider satisfaction are still incipient.

**Objective:**

This study aimed to describe the development, implementation, and expansion of a synchronous teleconsultation service targeting patients with symptoms of COVID-19 in Brazil, as well as to assess its usability and health care professionals’ satisfaction.

**Methods:**

This mixed methods study was developed in 5 phases: (1) the identification of components, technical and functional requirements, and system architecture; (2) system and user interface development and validation; (3) pilot-testing in the city of Divinópolis; (4) expansion in the cities of Divinópolis, Teófilo Otoni, and Belo Horizonte for Universidade Federal de Minas Gerais faculty and students; and (5) usability and satisfaction assessment, using Likert-scale and open-ended questions.

**Results:**

During pilot development, problems contacting users were solved by introducing standardized SMS text messages, which were sent to users to obtain their feedback and keep track of them. Until April 2022, the expanded system served 31,966 patients in 146,158 teleconsultations. Teleconsultations were initiated through chatbot in 27.7% (40,486/146,158) of cases. Teleconsultation efficiency per city was 93.7% (13,317/14,212) in Teófilo Otoni, 92.4% (11,747/12,713) in Divinópolis, and 98.8% (4981/5041) in Belo Horizonte (university campus), thus avoiding in-person assistance for a great majority of patients. In total, 50 (83%) out of 60 health care professionals assessed the system’s usability as satisfactory, despite a few system instability problems.

**Conclusions:**

The system provided updated information about COVID-19 and enabled remote care for thousands of patients, which evidenced the critical role of telemedicine in expanding emergency services capacity during the pandemic. The dynamic nature of the current pandemic required fast planning, implementation, development, and updates in the system. Usability and satisfaction assessment was key to identifying areas for improvement. The experience reported here is expected to inform telemedicine strategies to be implemented in a postpandemic scenario.

## Introduction

The COVID-19 pandemic has brought dramatic transformative changes in economies, societies, and health care, with an unprecedented challenge to public health worldwide [1]. The need to avoid patient crowds in health services and offer alternative ways for patient assistance while preserving physical distancing and isolation, as well as the prioritization of emergency departments and intensive care units, have proven to be important drivers for the urgent need and quick adoption of telemedicine.

Telehealth services were growing exponentially prior to COVID-19 [2]. However, it was during the pandemic that they received a major boost. Governments from different countries were urged to promote telehealth and make provisions to address some of the previously encountered barriers, and they quickly updated law restrictions and reimbursement policies [3]. In Brazil, telehealth has been consolidated over the years, but it was only after the spread of COVID-19 that a legal and regulatory framework emerged, authorizing remote medical and other professional health consultations. The Telehealth Network of the State of Minas Gerais (TNMG) in Brazil—one of the largest public telehealth services in Latin America [4,5]—was quick to implement telemedicine services for the care of patients with suspected novel coronavirus infection soon after the first patient was diagnosed with COVID-19 in the country.

Although a great number of teleconsultation services have been developed, studies assessing usability and satisfaction from the health care provider’s perspective are still incipient. Concerns have been raised regarding challenges posed by diagnosing without an actual physical examination and the negative impact on patient-provider rapport [6]. In the aftermath of COVID-19, when telehealth services are expected to remain in use and health care provider satisfaction is a key feature for telehealth sustainability, usability assessment is particularly relevant as a source to be tapped for lessons to be learned.

Our aim was to assess the feasibility of the development, implementation, and expansion of a synchronous teleconsultation service for care provided to patients with symptoms of COVID-19, as well as to perform assessments of usability and health care professionals’ satisfaction.

## Methods

### Study Design

**Figure 1:**
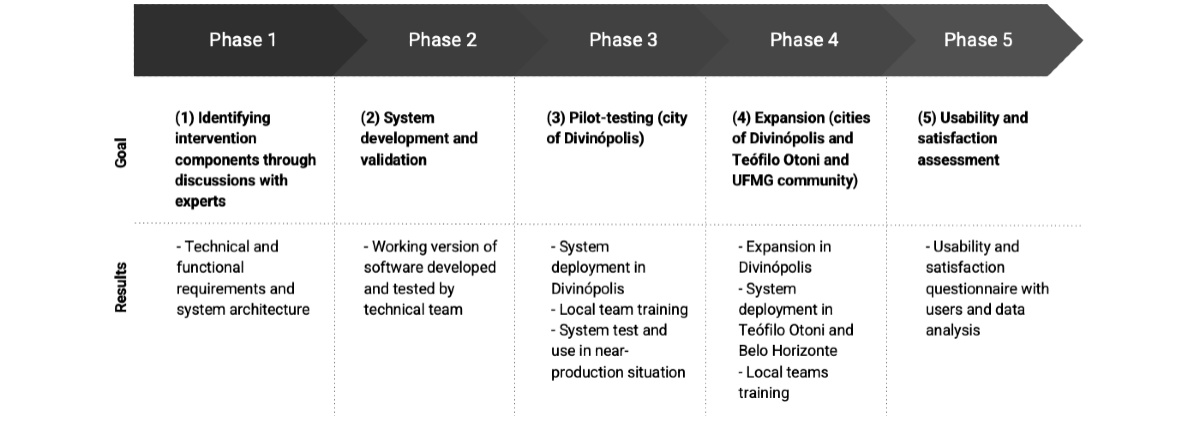
Project phases. UFMG: Universidade Federal de Minas Gerais.

This mixed methods study was developed in 5 phases (Figure 1)**,** following guidance from the Medical Council Framework [7]:

1. Identifying intervention components through discussions with experts;
2. System development and validation;
3. Pilot-testing;
4. Expansion; and
5. Usability and satisfaction assessment.

Each phase is briefly explained in the following subsections.

### Identifying Intervention Components Through Discussions With Experts

To identify components in the intervention, information was extracted from guidance issued by the Brazilian Ministry of Health [8,9] and evidence available at the onset of the pandemic, as well as discussions among an interdisciplinary team of IT specialists, clinicians with long-term expertise in telemedicine [4,5], infectious diseases specialists, and nurses.

The workflow suggested by the Brazilian Ministry of Health was adapted to improve the assistance flow, offer agility for the teams and data security, and reduce the burden of patients who need in-person consultations at primary care centers. The municipality where the system was planned to run initially—Divinópolis—had a telephone service dedicated for the general population to answer queries related to COVID-19 and for primary care practitioners, medical university professors, and undergraduate medical students working in a monitoring program. All available resources were used to design an integrated teleassistance flow, which assisted patients from their initial doubt through to clinical assistance and monitoring, at 4 levels: level 1, performed by local health care professionals (nursing technicians, physiotherapists, nutritionists, and psychologists); level 2, performed by nursing staff; level 3, performed by medical staff; and level 4, telemonitoring that was performed by students under medical supervision.

Although this teleassistance flow was not fully integrated into the local emergency departments, upon concluding teleconsultations, when face-to-face assessments were deemed necessary, the patients could be referred to face-to-face medical consultations with a specific clinical report.

An internal medicine specialist, a nurse, a doctor with long-term experience in telemedicine, and an IT specialist identified the main components in the intervention to map the main needs, steps in the process of care, and specificities of each screen and functionality in the system. These health care professionals worked alongside the IT specialist to discuss and propose changes and improvements to the system throughout the iterative development cycle adopted. Unfortunately, due to the necessary urgency of the actions—the platform was offered for use just 2 months after the start of its development—it was not possible to involve patients in the development of the self-assessment tools.

A management model based on the Plan-Do-Check-Act cycle and a monitoring system based on key performance indicators were developed. The nurse and the doctor with long-term experience in telemedicine defined the indicators to be monitored (Table S1 in Multimedia Appendix 1).

### System Development and Validation

The system was developed and validated following an agile software methodology. Its backend was built using the Java programming language (version 1.8) with the *Spring Boot* and *Hibernate* frameworks, whereas the system’s user interface was built using the *Angular* user interface framework.

The system, named TeleCOVID-MG, started being developed in March 2020. Throughout March and April, the team of analysts met weekly with the clinical team to assess new requests that arose. Meanwhile, the development team delivered weekly packages that were internally tested and, on weekends, were validated and approved by professionals from the clinical team. Thus, in May 2020, the first version was released into a production environment. From then on, fortnightly sprints were adopted, generating deliveries for testing and approval.

**Figure 2:**
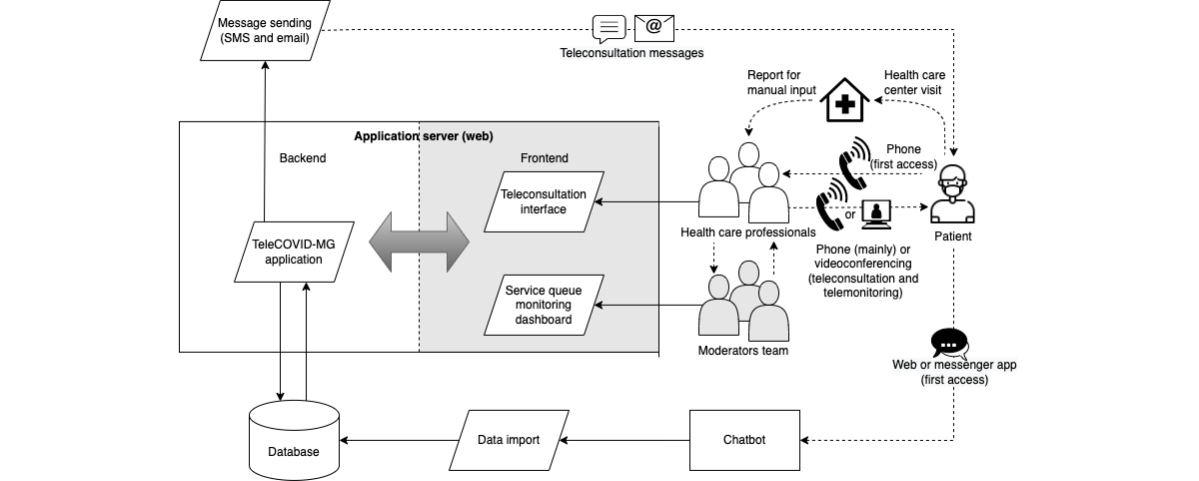
System architecture overview.

The software runs on a web environment, which allows the full recording of activities. It is composed of an application server, which runs the main application backend and serves the frontend to the users’ client browsers, and an SQL relational database (Postgres). The frontend has 2 main interfaces: 1 for teleconsultation, which is used by the health care professionals, and another for monitoring the service queue, which is a dashboard used by the team of moderators, who identify the need for additional health care professionals in the shift to reduce the response time. A chatbot, developed using the BLiP platform (Take), was aimed to be a first point of contact for patients with the telehealth service [10,11]. It assisted in screening the severity of respiratory and flu-like symptoms and queuing patients for teleconsultation based on warning-sign severity [10,11]. There is a module for importing data from the chatbot into the database and a module for sending messages to patients (Figure 2).

### Teleconsultation and Telemonitoring Services

**Figure 3:**
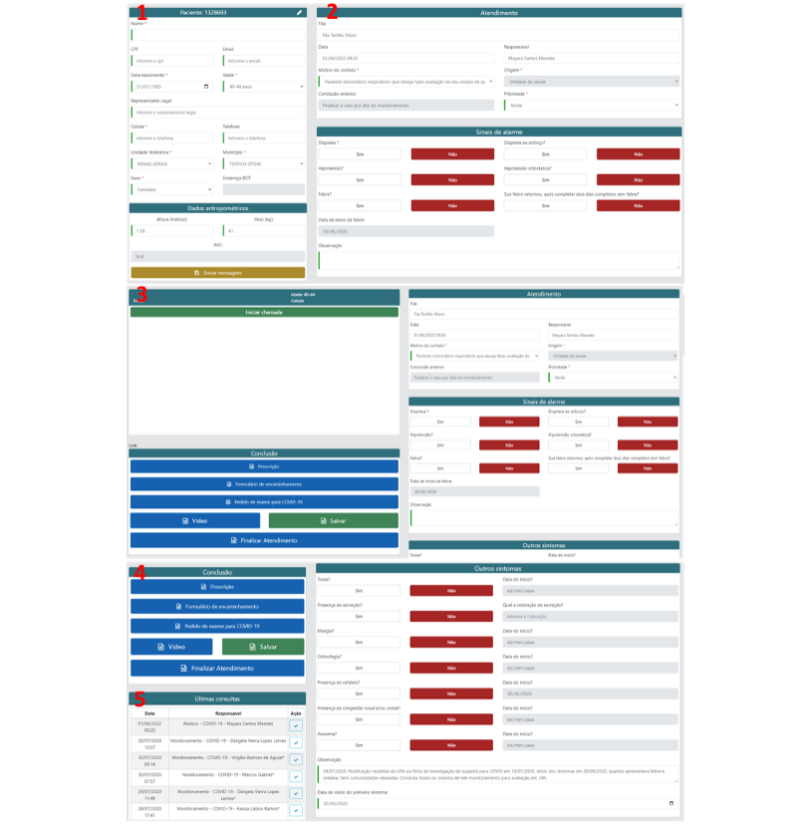
Screenshots of TeleCOVID-MG. User registration form: (1) patient personal information tab; (2) patient clinical condition tab recording warning signs; and (3) video call tab; (4) form tab (for prescriptions, reports, and test orders); and (5) record of past teleconsultations.

**Figure 4:**
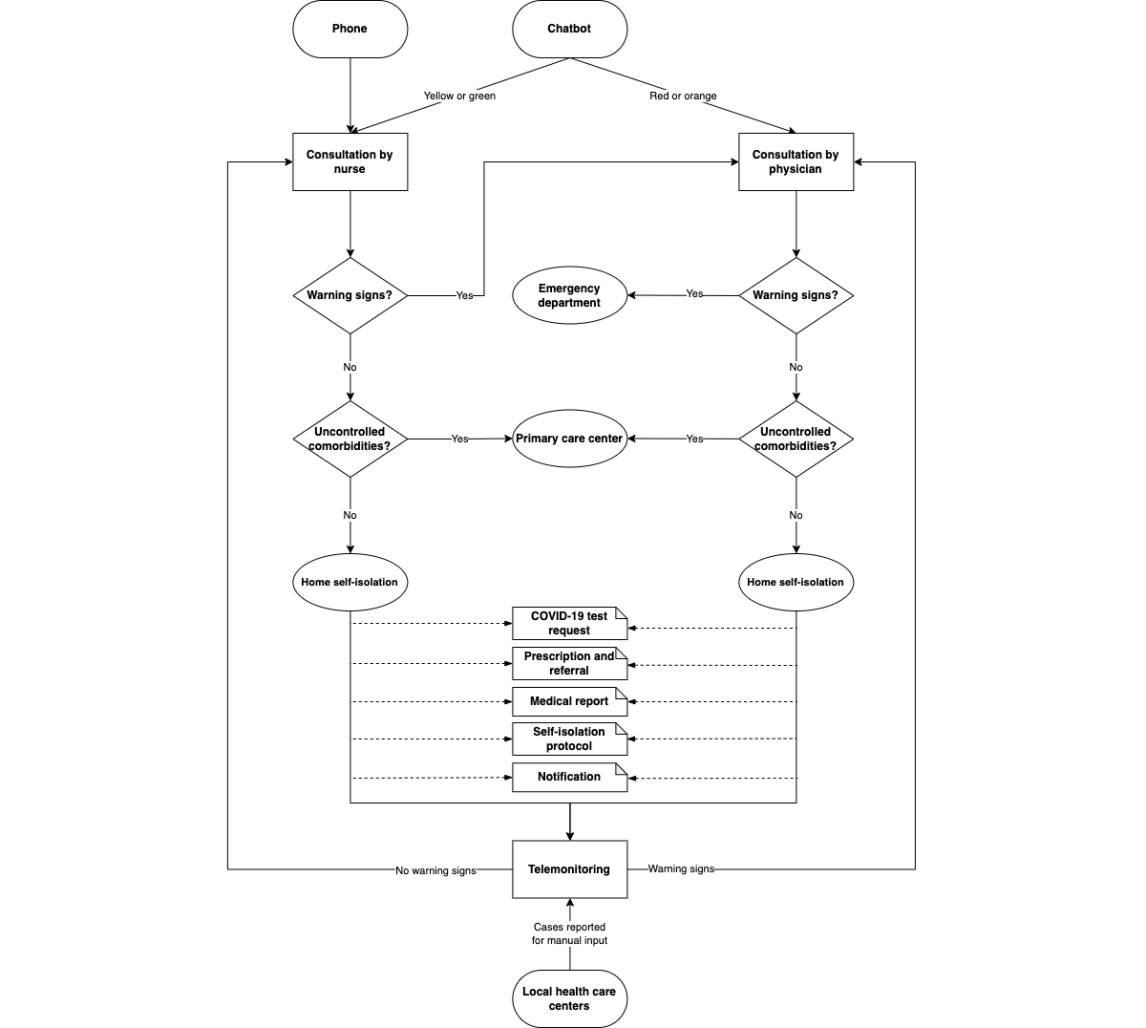
TeleCOVID-MG service workflow.

TeleCOVID-MG has 3 main goals: (1) assessing and managing patients with respiratory or flu-like symptoms, (2) monitoring patients with COVID-19, and (3) providing the general population with updated information about COVID-19. The system enables performing consultations either with or without videoconferencing, issuing medical prescriptions and reports, as well as issuing orders for diagnostic COVID-19 tests (Figure 3) by nurses and physicians from the TeleCOVID-MG teleconsultation team, following the Ministry of Health and local clinical protocols. All these documents generated during the teleconsultation can be easily downloaded as PDF files by the patients. The software also enables the generation of the compulsory report of COVID-19 cases, in compliance with requirements by the Brazilian Health Ministry, as well as teleconsultations scheduling, patient referral to telemonitoring services, or face-to-face consultations at other levels of care (Figure 4 and “TeleCOVID-MG service workflow” in Multimedia Appendix 1 [6,9,12]).

### Pilot-testing

The pilot study was carried out in Divinópolis, a 213,016-inhabitant city with a human development index of 0.76, from May 18 to 28, 2020 [12]. The team responsible for teleconsultations and telemonitoring in Divinópolis comprised physicians, nurses, and students from Universidade Federal de São João del-Rei. An instruction manual was prepared, and participating health care professionals received web-based clinical training, based on the best available scientific evidence at that time. They were also trained to use the system before starting the activities.

### Expansion

The project was expanded to the Teófilo Otoni, a 140,937-inhabitant city with a human development index of 0.70 [11], and subsequently to faculty and students at Universidade Federal de Minas Gerais (UFMG), a federal university where the coordination center of the TNMG is located. It has over 45,000 students and 7400 faculty members.

The team responsible for the teleconsultations comprised physicians and nurses from the TNMG, and, in the case of Teófilo Otoni, also included nurses from that city. The team responsible for telemonitoring comprised medical students supervised by medical professors and nurses. All of them received technical training to operate the system and theoretical training, as aforementioned. Weekly meetings were held with local coordinators to discuss indicators, identify deviations from planned targets, and plan and implement corrective actions.

Once patients entered the system, after the initial teleconsultation, a follow-up plan that involved monitoring or new consultations was defined based on the assessment of their situation. Teleconsultation efficiency was calculated as the number of patients who were provided with consultation and did not need to be referred to face-to-face consultations divided by the total number of patients who were provided with consultation.

For reporting expansion results, all records of patients who were provided with consultation at the 3 locations from May 2020 to April 2022 were eligible.

### Usability and Satisfaction Assessment

A questionnaire was developed to assess health care professionals’ satisfaction and the usability of the system, as they were the primary immediate users. It included eight 5-point Likert-scale questions that focused on aspects regarding user satisfaction and usability and open-ended questions that focused on the perceived strengths and weaknesses of the system, features to be improved, and comments about their experience with the system. All health care professionals who worked in the service and used the TeleCOVID-MG system were eligible (n=60). A thematic analysis was conducted for the open-ended questions.

### Ethics Approval

Ethical approval was obtained from the UFMG Research Ethics Committee (CAAE: 35953620.9.0000.5149). Informed consent was obtained from study participants.

## Results

**Figure 5:**
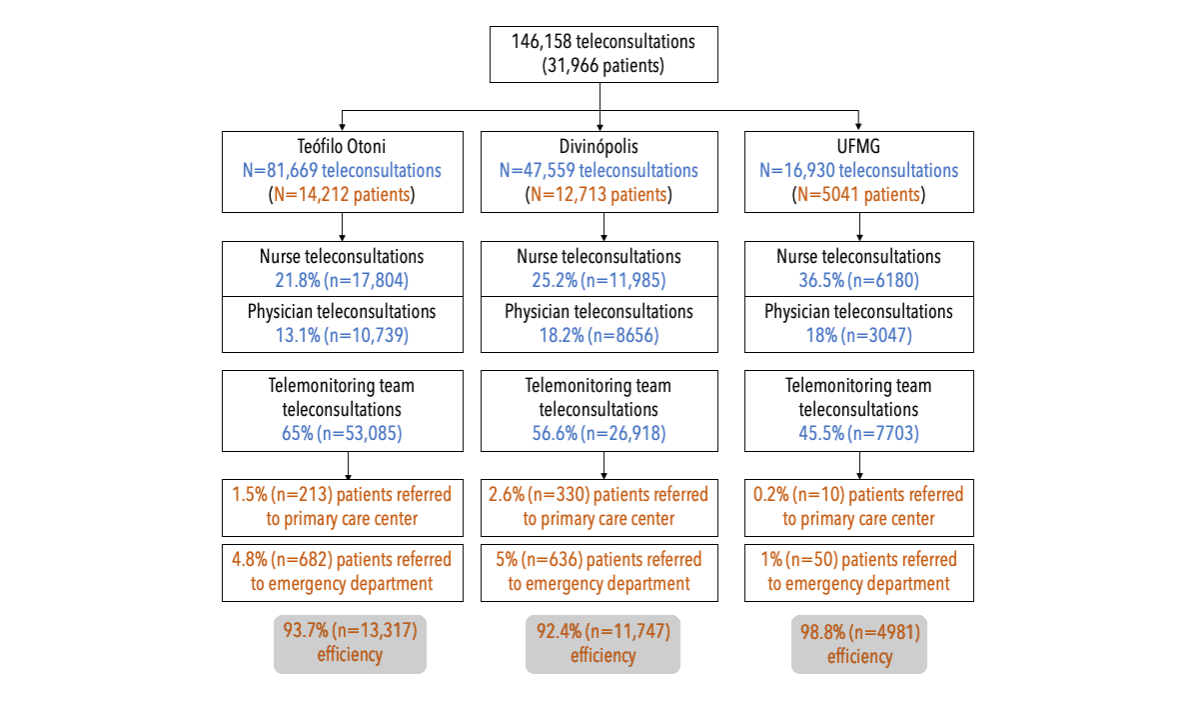
Percentage of teleconsultation distribution efficiency among the 3 cities in our study. UFMG: Universidade Federal de Minas Gerais.

Through the study, the system served 31,966 patients, totaling 146,158 teleconsultations covering the first and subsequent consultations performed for each patient, since the same patient could be assessed more than once by nurses, physicians, and the telemonitoring team. The accumulated number of teleconsultations and patients assisted by location and service efficiency are displayed in Figure 5. Other indicators that were monitored weekly and monthly are shown in Table S2 in Multimedia Appendix 1. The real-time analysis of these data allowed system and service workflow adjustments as necessary.

As shown in Figure 2, both the chatbot and telephone number were the gateway to the program. The telephone was primarily used, and teleconsultations were initiated through the chatbot in 27.7% (40,486/146,158) of cases. Additionally, the main method used to carry out the teleconsultations was via telephone call, with videoconferencing showing a very low usage rate (only 192 [0.13%] videoconferencing teleconsultations in total). When carried out, videoconferencing was performed via smartphone, using patients’ preferred software that was previously installed on their device. The main challenge faced during the expansion phase in Teófilo Otoni was the difficulty in reaching patients, even by telephone call, which may be due to the instability of the local telephone network and the fact that part of the population lives in rural areas and lack familiarity with telecommunication tools. Due to these same logistical and cultural reasons, the use of videoconferencing and other technologies such as chatbot was even more challenging.

Another difficulty was aligning the clinical guideline developed for remote care with the practice carried out in the city. To face this challenge, training meetings were held with local health teams, and several seminars addressing theoretical issues related to the management of patients with COVID-19 were carried out. The number of assessments initiated via chatbot was low in Teófilo Otoni, which may be due to the low socioeconomic level of the population and their limited digital literacy when dealing with new technologies. However, the number of calls via chatbot was extensive among the university community at UFMG, which is consistent with college users who have the digital skills needed to deal with chatbots.

Of the 60 health care professionals who used the system when the assessment was performed, 50 (83%) answered the questionnaire (age: median 35, IQR 31-40 years; women: n=43, 86%). Of these professionals, 42% (n=21) were physicians and 54% (27) were nurses (Table S3 in Multimedia Appendix 1).

Overall, the system was evaluated as satisfactory (Table 1 and Figure S1 in Multimedia Appendix 1). The only exception was for the statement “The system is stable, and no errors occur during use,” which had a median score of 4 (IQR 2-4). We believe this score reflects technical infrastructure problems and the short time taken to put the system into production, which prevented debugging. With regard to the open-ended questions, 35 participants answered at least one question, 44 commented on the system’s strengths, 41 mentioned weaknesses, 37 made suggestions, and 16 commented on their experience with the system (see “Responses to the open-ended questions” in Multimedia Appendix 1).

**Table 1:** Usability and satisfaction assessment (n=50). Likert-scale responses range from 1 to 5: 1=totally disagree, 2=partially disagree, 3=indifferent, 4=partially agree, and 5=totally agree.

Item	Median (IQR)	Mean
System screens can be easily understood.	5 (5-5)	4.9
The system allows recording all relevant data on patient consultation.	5 (4-5)	4.64
By following the screen prompts, I was able to provide patient care with quality.	5 (5-5)	4.86
The system fields are easy to fill out.	5 (5-5)	4.84
The system is intuitive to use.	5 (4-5)	4.42
I believe that the system can be useful in clinical practice, for the care of patients with suspected COVID-19.	5 (5-5)	4.96
The system is stable, and no errors occur during use.	4 (2-4)	3.3
I was satisfied with the use of the system.	5 (4-5)	4.64

## Discussion

### Principal Findings

Our study presents a novel telehealth tool from its conceptualization through its development, validation, implementation, and rapid expansion. When planning the teleconsultation system, 2 major barriers to the implementation were identified. First, the lack of local experience with the functionalities needed for synchronous teleconsultation. Despite the TNMG’s long experience with other telehealth tools, it was only after the spread of COVID-19 that a legal and regulatory framework emerged, authorizing remote medical and other professional health consultations in Brazil [13]. Second, as COVID-19 was a new disease, information about it was still scarce. Due to the successive emergence of new evidence, the system’s initial matrix had to be progressively changed over time. Thus, a continuous development and validation process was of utmost importance to guarantee that the system was kept in line with updated evidence.

The use of the TeleCOVID-MG system made it possible to clarify queries about the novel coronavirus and deliver remote care to thousands of patients, thus reducing the circulation of individuals with respiratory or flu-like symptoms, minimizing the burden on health services, and increasing patient access to care in places with scarce health resources; together, these possibilities evidence the critical role of telemedicine in expanding emergency services capacity during a pandemic. The system also contributed to the updating of several health care professionals on the main topics related to COVID-19.

Doubts and concerns about the use of teleconsultations, especially teleconsultations performed by telephone calls (which was the most frequently used medium in our context), were already present even before the pandemic. Impossibility to perform the physical examination, compromised physician-patient relationship, difficulty in performing a global assessment of the patient that only focus on acute complaints, and uncertainty in the quality of information are some of the challenges reported in studies that evaluated the perception of health care professionals about the use of teleconsultations [14-17]. With the pandemic, providers and patients were forced into a new normal that included communicating with each other through video and audio [18], and studies have demonstrated that the COVID-19 pandemic affected the way physicians use and perceive telehealth and increased telehealth activities use both in type and frequency [19] despite the aforementioned limitations. Through their experience during the pandemic, physicians became more convinced of the efficacy, efficiency, and safety of telemedicine, as well as their ability to meet their patients’ needs remotely. Although there was a shift in physicians’ activities and perceptions, concerns about the effectiveness of remote consults and the lack of adequate legal frameworks remain [17]. Negative aspects related to teleconsultations reported in the literature include concerns about the absence of visual clues, inability to perform a physical examination, and thus the lack of comprehensive assessments [17,18].

Health care professionals with no experience in telehealth needed to quickly develop skills in web-based rapport building [19]; therefore, assessing the usability and provider satisfaction of each implemented system is of utmost importance. The analysis of usability and satisfaction of health care professionals with our system showed that most of them agreed that the system is intuitive and easy to understand and operate; allows them to provide care with quality; and is useful for evaluating patients with COVID-19. The social function of the systems was highlighted for the way it guaranteed the expansion of access to health care and decreased the burden in local health care. In addition, the systems allow interdisciplinarity and the development of a continuum of care until the patient’s complete recovery.

Our results are in line with other studies [4,20-22], which showed high levels of satisfaction with telemedicine implemented during the pandemic. A recent integrative review has found 5 studies assessing provider satisfaction, all of them in outpatient clinics for specialized care during the pandemic for other conditions, and satisfaction ranged from 78% to 93% among the studies [23]. The evidence presented here suggests the feasibility of incorporating synchronous teleconsultations for the management of other health conditions. For this application to be possible, we emphasize the need for constant improvements in the systems and the importance of integrating remote care with face-to-face care.

Bearing in mind that the uptake and sustainability of telehealth interventions are the ultimate goals when implementing them, we highlight the following as takeaway lessons:

- Previous expertise is important for the successful development of a new system, particularly when implementation within a short amount of time is needed;
- The engagement of end users, in this case health care professionals, in system design and development is of utmost importance to ensure the fulfillment of user needs and usability;
- Health care professionals’ perception of telehealth was positively impacted by the pandemic setting, as shown by their reported high levels of satisfaction; and
- In remote or resource-constrained locations with unstable internet, having an alternative way to perform teleconsultation (such as using telephone calls) is of utmost importance.

The main challenges faced in the usability of the TeleCOVID-MG system were related to the instability of the local telephone network, the need to align the clinical guideline developed for remote care with the practices carried out in the municipalities, and continually adjusting the system to the new scientific evidence and practices arising through the course of the pandemic.

As limitations of the TeleCOVID-MG system, we should remark that the lack of integration with data from face-to-face assistance were reported. The need for an interoperable health care system became blatantly evident worldwide during the COVID-19 pandemic to avoid duplicating work and improve decision-making. Although not designed for interoperability, the system architecture allowed the on-demand generation of customized queries and reports.

With great growth in the use of teleconsultations as a way to fight the pandemic, several entities have published guidelines to help health care professionals in remote patient care [24]. Furthermore, studies have been published focusing on evaluating the use of this telehealth tool and proposing adjustments for expansion in the postpandemic period [18,20].

### Limitations

With regards to the efficiency assessment, although the team performed a thorough assessment of referrals and nonreferrals, there might be cases in which patients did seek face-to-face care despite not having been recommended to do so. Patient and caregiver experience, as well as patient digital literacy and satisfaction with the TeleCOVID-MG service, has not been formally addressed yet. We opted to restrict our analysis to health care professionals due to 2 main reasons: (1) they had to adapt their work routine very quickly due to the pandemic; and (2) they were the primary users of the teleconsultation system, as they had to fill out the patients’ electronic record and issue medical prescriptions, reports, and orders for diagnostic COVID-19 tests through the system. Despite the lack of formal assessment with patients, the assisting health care professionals reported spontaneous comments from patients on how they felt welcomed and listened to in a better way than in face-to-face consultation, as they had time to report everything they wanted, without the time constraints present in face-to-face consultations. This finding supports the idea that it is indeed possible to provide humanized care in telehealth. We are currently conducting a formal patient satisfaction analysis for TeleCOVID-MG.

Due to the pandemic scenario and the goal of including as many health care professionals who were using the system as possible in our usability and satisfaction assessment, our analysis was centered mainly on our questionnaire. A more thorough analysis about satisfaction drivers using a more in-depth qualitative study could provide additional lessons.

### Conclusion

This paper described the rapid development, implementation, and expansion of the TeleCOVID-MG system, as well as the results of our usability and satisfaction assessment with health care professionals. The system made it possible to answer queries about COVID-19 and provide remote care to thousands of patients, showing the critical role of telemedicine in expanding emergency services capacity during a pandemic. The dynamic nature of the current pandemic required regular updates in the system and frequent monitoring of the implemented actions. The experience reported here is expected to inform telemedicine strategies to be implemented in a postpandemic scenario, not only to deal with eventual new pandemics but also, and most importantly, explore the affordances of telemedicine to enhance public policies aimed at promoting health care prevention, treatment, and education. Furthermore, our experience illustrates the local and cultural challenges and specificities that need to be dealt with in the development of such systems, which indicate that even if there were “off-the-shelf” solutions available, they might not be able to address local community needs.

## Appendix

Multimedia Appendix 1 Supplementary file.

## Abbreviations

TNMG: Telehealth Network of the State of Minas Gerais
UFMG: Universidade Federal de Minas Gerais

## Data Availability

Data is available upon reasonable request.
