# Supplementary material for: Synchronous Teleconsultation and Monitoring Service Targeting COVID-19: Leveraging Insights for Postpandemic Health Care
Source: JMIR Med Inform. 2022 Dec 22;10(12):e37591. doi: 10.2196/37591 (PMC9786675; doi:10.2196/37591)
Supplement: Multimedia Appendix 1 [file medinform_v10i12e37591_app1.docx]

**MULTIMEDIA APPENDIX 1**

**1. Table S1** – Indicators …………………………………………………………….p.2

**2. TeleCOVID-MG service workflow**……………………………………………..p.4

**3**. **Table S2** – Summary of indicators data………………………………………….p.6

**4. Table S3** - Sociodemographic data of healthcare professionals participating in the study………………………………………………………………………………….p.7

**5. Figure S1.** Boxplot of response distributions of usability and satisfaction questionnaire………………………………………………………………………..p.8

**6. Responses to the open-ended questions in usability and satisfaction assessment**…………………………………………………………………………...p.9

**1. Table S1** – Indicators

| **12 SELECTED INDICATORS** | 1 – Number of monthly TeleCOVID-MG teleconsultations (levels I, II, III & IV); |
| --- | --- |
|  | 2 – Number of monthly TeleCOVID-MG patients (levels I, II, III & IV); |
|  | 3 – Average monthly teleconsultations per hour and day at levels I, II, III & IV; |
|  | 4 – Number of monthly non-effective patient contacts; |
|  | 5 – Number of monthly patients not agreeing with terms of use; |
|  | 6 – Percentage of monthly patients agreeing with TeleCOVID-MG terms; |
|  | 7 – Average teleconsultation time at levels I, II, III & IV; |
|  | 8 – Number of monthly completed cases at level II & III; |
|  | 9 – Number of monthly patients kept under monitoring at level IV; |
|  | 10 – Number of monthly patients discharged at level IV; |
|  | 11 - Percentage of monthly TeleCOVID-MG solved cases; |
|  | 12 – Number of monthly patients referred to Basic Health Centers or emergency units. |
| **PRODUCTION** | Number of monthly TeleCOVID-MG teleconsultations (levels I, II, III & IV); |
|  | Number of monthly TeleCOVID-MG patients (levels I, II, III & IV); |
|  | Average monthly teleconsultations per hour at levels I, II, III & IV; |
|  | Average monthly teleconsultations per day at levels I, II, III & IV; |
|  | Number of monthly teleconsultations (levels I, II, III & IV). |
| **MANAGEMENT** | Number of monthly non-effective patient contacts; |
|  | Number of monthly patients not agreeing with terms of use; |
|  | Average teleconsultation time at levels I, II, III & IV; |
|  | Number of monthly finalized cases at level II & III; |
|  | Number of monthly patients kept under monitoring at level IV; |
|  | Number of monthly patients discharged at level IV; |
|  | Number of monthly patients referred to Basic Health Centers or emergency units. |
| **MEDIA REPORTING** | Number of monthly TeleCOVID-MG teleconsultations (levels I, II, III & IV); |
|  | Number of monthly TeleCOVID-MG patients (levels I, II, III & IV); |
|  | Percentage of monthly patients agreeing with TeleCOVID-MG terms; |
|  | Percentage of monthly TeleCOVID-MG resolved cases; |
|  | Number of monthly patients referred to Basic Health Centers or emergency units. |

**2. TeleCOVID-MG service workflow**

User access is either via chatbot [9] or local phone call as a dedicated service (Figure 4). It is also possible for local managers to include patients that were face-to-face assisted in the municipality with respiratory complaints. Screening is performed through a list of questions elaborated drawing on the best available evidence. This first interaction is conducted by the chatbot agent or by professionals trained for the task (level 1). After screening and according to the severity of symptoms and comorbidities, patients are assessed by a nurse or a physician (levels 2 and 3).

Patients classified with a “red” or “orange” tag following criteria for emergency care established by the World Health Organization (WHO) and the Brazilian Health Ministry are assessed directly by medical staff (level 3) [6,12]. The physician confirms whether warning signs are present or not and, if that is the case, refers the patient to onsite evaluation at the emergency service.

Patients classified with a “yellow” tag, because they have comorbidities, though do not report any warning signs, are assessed by nursing staff (level 2). After an initial evaluation, nurses can advise the patient to seld isolate at home, to seek onsite evaluation at a primary care center, or request being assessed by the medical staff when needed (level 3).

Patients classified with a “green” tag, as they show respiratory tract symptoms without any warning signs and have no comorbidities, are also assessed by nursing staff (level 2). In this case, nurses can advise the patient to keep domiciliary isolation, or refer them for evaluation by the medical staff when needed (level 3).

All patients evaluated through the teleconsultation system, regardless of the severity of their symptoms, the presence of comorbidities, and the advice given by the medical or nursing teams, are included in the telemonitoring program (level 4), which monitors and provides support to them for at least ten days after the onset of respiratory symptoms. Undergraduate medical students specifically trained and working under the supervision of a physician or a nurse make up the telemonitoring team. Telemonitoring is performed through a phone call performed every 48 hours, or every 24 hours if the patient is 60 years old or above or if the patient has a decompensated comorbidity. The students check symptoms and, if any warning sign or other complication emerges during the period of follow-up, they request an evaluation by the nursing or medical staff.

**3. Table S2** – Summary of indicators data

**4. Table S3** - Sociodemographic data of healthcare professionals participating in the study

| **Variable** | **N=50** |
| --- | --- |
| **Professional training**  Physician  Nurse  Physical educator  Psychologist | 21 (42,0)  27 (54,0)  1 (2,0)  1 (2,0) |
| **First degree obtained (years ago), median (IQR)** | 13 (11-15) |
| **Work field**  Primary care  Ambulatory  Hospital service  Lecturing | 28 (56,0)  9 (18,0)  7 (14,0)  6 (12,0) |
| **Number of times system was used**  Fewer than 5  06 to 10  11 to 20  Over 20 | 3 (6,0)  1 (2,0)  6 (12,0)  40 (80,0) |

**5. Figure S1**. Boxplot of response distributions of usability and satisfaction questionnaire


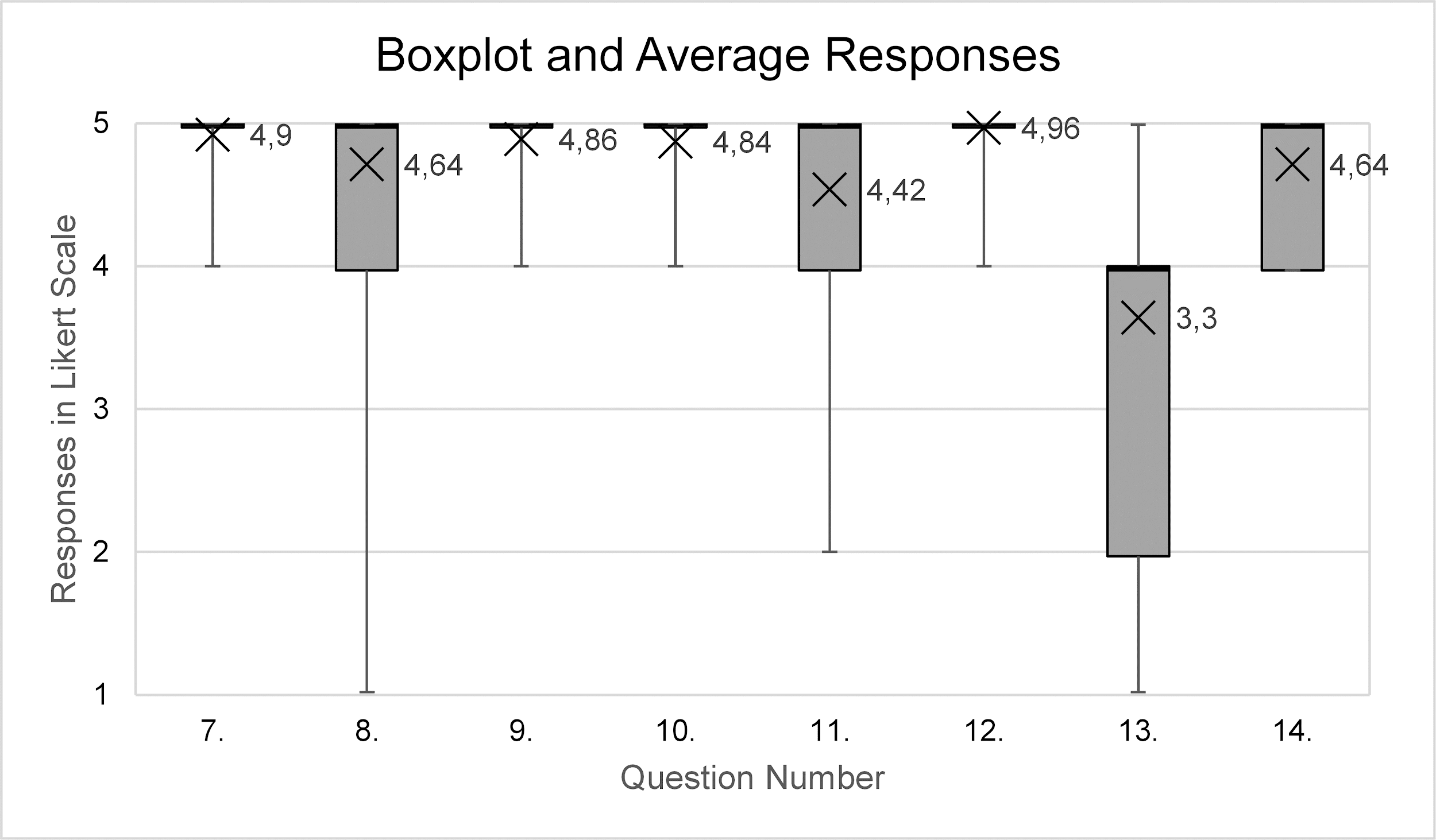


**6. Responses to the open-ended questions**

Regarding weaknesses, participants reported on three main problems: system robustness; interaction design decisions, and medically related design decisions. The great majority of the comments regarding weaknesses pertain to system robustness, that is, the system being unstable or having bugs. P31, for instance, stated that “*previously issued medical prescriptions/reports previously issued were not always saved”.* This is echoed b*y* P44, who reported “*errors in recording data and sending documents”.* Participants ascribed some of the problems experienced to available infrastructure rather than to the system itself. For instance, P11 blamed Internet connection instability for “*errors in information* *saving* [...]” and P9 suggested improving “*technological infrastructure to avoid errors and instability”*. These reports are in line with the results regarding system stability in the quantitative analysis.

Participants also raised issues regarding design decisions, which we have classified into two groups: those specific to interactive aspects of the system, and those that pertain to medical aspects. Design decisions regarding interactive aspects included the size of fields in forms, feedback issues, help information, as well as the need for some specific user actions, such as explicitly saving the forms and having to download prescriptions to sign them. For instance, P33 suggested that “*There should be some way for the system to confirm that a report has been sent. Reports are saved in consultation [system tab], but there is no confirmation message.”* and P16 pointed out the disadvantage that there is no “*automatic saving mode.”* It is worth highlighting that comments on weaknesses and improvement suggestions are sparse, no point was cited by more than one participant.

As for design decisions regarding medical aspects, there are comments that focus on operational aspects, whereas others refer to more strategic aspects of the solution implemented in the system. Operational aspects refer to options for a particular form field or to those who should be able to edit information. For instance, a participant mentioned that the field “"reason for consultation"” should have a more encompassing list of options to choose from. Regarding the more strategic aspects, there were some comments about different paths that lead patients to the queue. P24 thought that patients screened with a red code by the chatbot should not be handled as online consultation, as they would require a face-to-face consultation.

Finally, some comments were exceeding the particular experience of teleconsultation. P17 suggested that the system should generate reports on the number of recovered patients, and P36 thought it a shortcoming that TeleCOVID-MG was not integrated into local health services.

Most of the suggestions made by participants pertain to the need to improve the weaknesses mentioned above. Some participants, though, suggested improvements and extensions to the system. P33 suggested that “*The medical report screen should be automatically filled in with the data of the patient under care [...]”* while P42 thought it important to “*[...] Improve the search engine to find similar words in the database; make the system calculate age automatically when the date of birth is entered; allow for more than one reason for the call to be registered at the level I, remove zip code as mandatory data,[...]”.* Participants also made suggestions for new functions within the goals of the system such as improving the search engine by making it possible to assess the list of consultation records per healthcare professional. They also made suggestions that would involve broadening these goals, such as allowing for communication through the system of professionals regarding a patient's case or between teams in different shifts, including support for clinical decisions and integrations with other health services being offered to the community.

Regarding the system's strengths, the great majority of the participants mentioned that the system is easy to understand and use, being both efficient and useful. They also mentioned that the system collects a lot of relevant information about the patient and systematizes anamnesis. These points can be illustrated by P35's response: “*intuitive, clear and clean visual appearance, quick to fill in.*”, and P28's response: “*easy to understand and containing all relevant information for patient assessment.*” They also mentioned that the system supports patients (P21: “*The advantages are to promote quality care at a distance and to facilitate patient access to test orders, prescriptions, and reports*.”) and its relevance during the pandemics (P26: “*promotes access to health care safely and comfortably for the user, who is cared for in his/her living environment and telemonitored, with continuity of care until recovery. Greater control of contacts of suspected or confirmed cases with precise information to reduce the chain of transmission, by maintaining social distance through rapid access to medical reports necessary for social isolation.”*).

Although participants pointed out weaknesses (the main one being the system's stability) and aspects to be improved, overall their comments indicated that they were satisfied with the system, and considered their experience with the system a positive experience. P36 expressed this view in her comment about her experience with the system “*Overall I find the application fantastic and have many more compliments than complaints*.”.
